# Radiation dose reduction in thoracic and abdomen–pelvic CT using tube current modulation: a phantom study

**DOI:** 10.1120/jacmp.v16i1.5135

**Published:** 2014-01-08

**Authors:** Akmal Sabarudin, Zakira Mustafa, Khadijah Mohd Nassir, Hamzaini Abdul Hamid, Zhonghua Sun

**Affiliations:** ^1^ Diagnostic Imaging & Radiotherapy Programme Universiti Kebangsaan Malaysia 50300 Kuala Lumpur Malaysia; ^2^ Radiology Department Universiti Kebangsaan Malaysia Medical Centre Kuala Lumpur Malaysia; ^3^ Department of Imaging and Applied Physics Discipline of Medical Imaging, Curtin University Perth Australia

**Keywords:** computed tomography, tube current modulation, radiation dose, dose reduction

## Abstract

This phantom study was designed to compare the radiation dose in thoracic and abdomen–pelvic CT scans with and without use of tube current modulation (TCM). Effective dose (ED) and size‐specific dose estimation (SSDE) were calculated with the absorbed doses measured at selective radiosensitive organs using a thermoluminescence dosimeter‐100 (TLD‐100). When compared to protocols without TCM, the ED and SSDE were reduced significantly with use of TCM for both the thoracic and abdomen–pelvic CT. With use of TCM, the ED was 6.50±0.29 mSv for thoracic and 6.01±0.20 mSv for the abdomen–pelvic CT protocols. However without use of TCM, the ED was 20.07±0.24 mSv and 17.30±0.41 mSv for the thoracic and abdomen–pelvic CT protocols, respectively. The corresponding SSDE was 10.18±0.48 mGy and 11.96±0.27 mGy for the thoracic and abdomen–pelvic CT protocols with TCM, and 31.56±0.43 mGy and 33.23±0.05 mGy for thoracic and abdomen–pelvic CT protocols without TCM, respectively. The highest absorbed dose was measured at the breast with 8.58±0.12 mGy in the TCM protocols and 51.52±14.72 mGy in the protocols without TCM during thoracic CT. In the abdomen–pelvic CT, the absorbed dose was highest at the skin with 9.30±1.28 mGy and 29.99±2.23 mGy in protocols with and without use of TCM, respectively. In conclusion, the TCM technique results in significant dose reduction; thus it is to be highly recommended in routine thoracic and abdomen–pelvic CT.

PACS numbers: 87.57.Q‐, 87.57.qp, 87.53.Bn

## I. INTRODUCTION

Computed tomography (CT) has become a routine imaging modality for many clinical applications due to its wide availability, less invasiveness, short scanning time, excellent anatomical resolution, and high diagnostic value.^(1)^ It is also suitable for patients who are contraindicated for magnetic resonance imaging (MRI) procedures, such as those with implanted metallic medical devices or pacemakers^2^, ^3^ and patients on ventilation with non‐MRI compatible oxygen tanks.

Previous studies have shown that CT has high sensitivity and specificity for detecting ureteric calculus with a 92% and 99% success rate, respectively.^(4)^ Furthermore, CT has been reported to be highly sensitive in the detection of bone metastases, with a success range of 71%–100%.^(5)^ The reported sensitivity, specificity, positive predictive value (PPV), and negative predictive value (NPV) of CT were 82%, 99%, 64%, and 97% for detection of bowel and mesenteric injuries^(6)^ and 100%, 95%, 97%, and 100% for diagnosis of appendicitis, respectively.^(7)^

The current CT scanners are able to acquire up to 640 image slices in a single gantry rotation, and they have been proven to give patients a lower radiation dose compared with previous CT generations. However, the low radiation dose associated with the latest CT scanners still remains higher than that received from other diagnostic imaging examinations. Therefore, radiation dosage has become a major issue in the medical literature as there is increased concern about radiation‐induced malignancy, which is especially important for pediatric patients undergoing CT scans. It has been reported that 70% of the radiation dose received by patients during medical procedures are from CT scans.^(8)^ Moreover, nearly half of all medical radiation to patients comes from CT, with the cumulative effective dose (ED) from CT imaging reported to be approximately 440,000 person‐Sieverts, which translates into an ED of nearly 1.5 mSv per capita.^(9)^ Thus, radiation‐induced malignancy is estimated to be the dominant cause of cancer mortality from full body CT examinations.^(10)^ It has also been reported that there is a significant correlation between organ doses and repeated CT examinations (two or three scans resulting in a dose range of 30 mSv to 90 mSv), which significantly increases cancer risk.^(11)^

Different dose‐reduction strategies have been developed.^(12)^ Of these strategies, tube current modulation (TCM) represents an effective method to reduce the radiation dose the patient receives by reducing X‐ray exposure in certain tube positions or projection angles along the patient's body. It is done entirely by determining the suitable tube current with localizer radiographic projection of the patient.

Using a TCM protocol, the tube current is adjusted automatically based on object thickness during each gantry rotation. This mode allows photons to pass through the object in a nonuniform way, with each part receiving a sufficient amount of photons to penetrate the patient without giving an unnecessary dose to the narrower body part, hence reducing the ED that patient receives. The TCM technique has been shown to reduce the radiation dose significantly, by up to 50%.^(12)^ In addition, automatically adjusted mA in both x‐, y‐ and z‐planes has been proven to produce CT images with consistent image quality in patients of different sizes.^(13)^ Furthermore, a combination of angular and z‐axis modulation scanning techniques can further reduce the radiation dose by up to 60% in abdomen–pelvic CT examinations.^(14)^ The radiation dose at radiosensitive organs (the breast and lungs) can also be reduced with the TCM technique in thoracic and abdomen–pelvic CT imaging without compromising image quality.^15^, ^16^

This phantom study was performed to investigate the radiation doses received in thoracic and abdomen–pelvic CT protocols focusing on ED, size‐specific dose estimation (SSDE), and absorbed dose in selected radiosensitive organs. This study is expected to provide valuable information to medical radiation practitioners, especially radiographers, to improve their practice when performing routine CT examinations.

## II. MATERIALS AND METHODS

**Figure 1:**
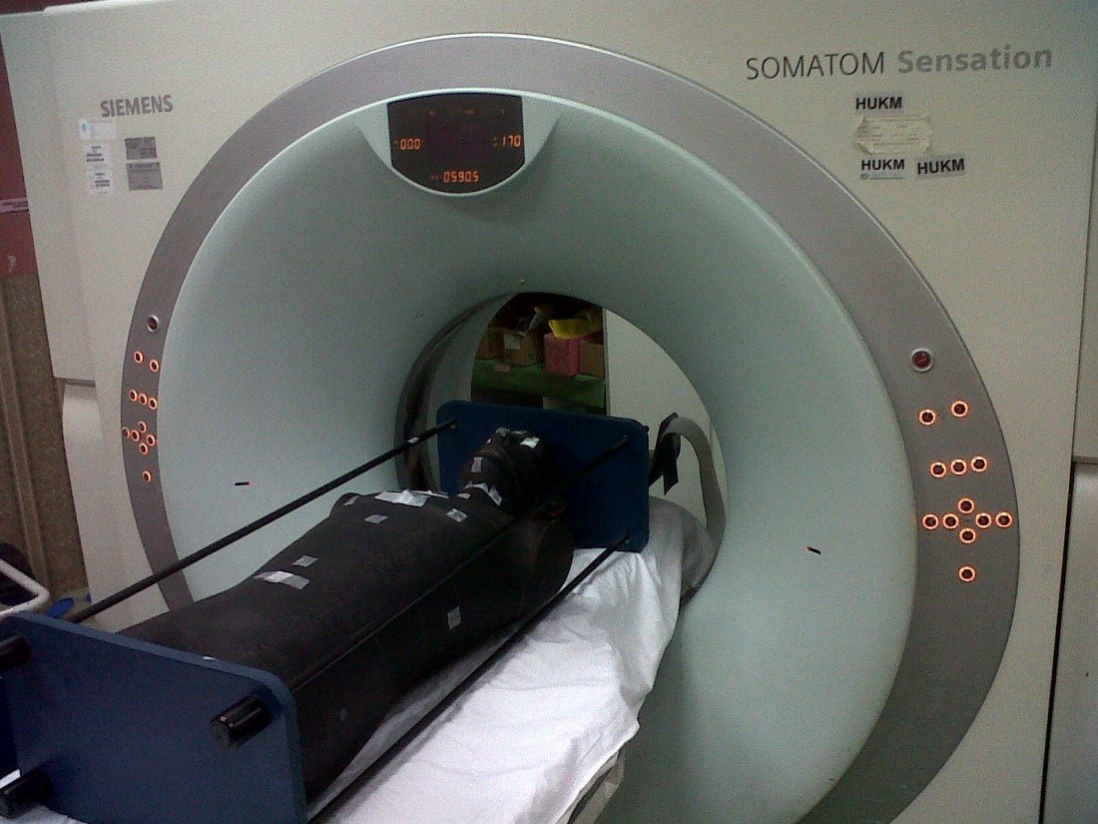
Image showing that the TLDs were securely placed on the skin surface of the anthropomorphic male Alderson RANDO phantom at the targeted radiosensitive organs.

This experimental study was performed on an anthropomorphic adult male Alderson‐Rando phantom (The Phantom Laboratory, Salem, NY) which mimicked an adult with 73.5 kg in weight, 175 cm in height, and made from tissue‐equivalent materials (Fig. 1). The phantom was made up of 36 slabs numbered from head to thigh and specifically designed to measure radiation doses using TLD chips with each slice (slab) of the body having small holes that fit the TLD. In this study, absorbed dose of the selected radiosensitive organs was measured, including gonad, bone marrow, lungs, colon, stomach, breast, urinary bladder, liver, esophagus, thyroid, kidneys, and skin.

The CT examination procedures were carried out using a multidetector CT scanner Somatom Sensation 64 (Siemens Medical Solutions AG, Munich, Germany). In addition, a dedicated low radiation exposure protocol, the CARE Dose4D, also known as the TCM technique, was used in this CT system. Results using this technique were compared to results from the standard protocol without use of TCM. All exposure parameters (kVp and mAs) were manually adjusted based on phantom size.

### A. Computed tomography protocols

#### A.1 Thoracic CT protocols

An iodinated contrast medium was not introduced to the CT scans on the anthropomorphic RANDO phantom due to the limitation of it not containing normal anatomical blood vessels. In this study, both CT protocols (with and without use of TCM) were used with the manual exposure parameter set at 120 kVp and 317 mAs for thoracic CT protocol without TCM, while the CARE Dose4D system was used in the TCM protocol (Table 1). The protocols were selected and modified according to those applied in the department's routine practice. In addition, selection of the parameters was based on previous studies in which recommendations were made to effect a significant dose reduction as a dose‐saving strategy in CT examination procedures.^12^, ^15^, ^16^ The topography scan was performed first for scan guidance, which includes the range from the chin to the iliac crest with the X‐ray tube positioned at 180° (below the couch). The scan was followed by helical acquisition of the thoracic cavity covering from the apex of the lungs to the dome of the diaphragm, which includes the lungs and axillary lymph nodes. The lateral CT laser was localized at the midcoronal plane of the phantom to maintain the x‐y location for the organ dose measurements.

**Table 1:** Thoracic and abdomen–pelvic CT protocols

	*Thoracic CT*		*Abdomen–Pelvic CT*	
*Parameters*	*With TCM*	*Without TCM*	*With TCM*	*Without TCM*
Collimation (mm)	64×0.6	64×0.6	64×0.6	64×0.6
Scanning mode	Helical	Helical	Helical	Helical
Slice thickness (mm)	5.0	5.0	10	10
Pitch	0.8	0.8	0.9	0.9
Rotation time (s)	0.33	0.33	0.33	0.33
Tube voltage (kV)	120	120	120	120
Tube current time (mAs)	∼185	317	∼192	300
Scan time (s)	12.07	12.05	12.69	12.66
Scan length (mm)	389.0	389.0	410.0	410.0
Orientation	Head first	Head first	Head first	Head first

TCM=tube current modulation.

#### A.2 Abdomen–pelvic CT protocols

As with the thoracic CT protocol, no iodinated contrast medium was introduced during these protocols (Table 1). Two acquisition series were performed, including topography and helical acquisition, starting from the dome of the diaphragm to the pubic symphysis, which includes the adrenal gland and gonadal organ. The exposure parameters kVp and mAs were set at 120 kVp and 300 mAs for the protocol without TCM, and the CARE Dose4D system was used for the TCM protocol.

### B. Radiation dose measurements

#### B.1 Absorbed dose

A high sensitivity thermoluminescent dosimeter‐100 (TLD‐100) (Thermo Scientific, Waltham, MA) containing lithium fluoride doped with magnesium, copper, and phosphorous (LiF:Mg,Cu,P) was used to measure the absorbed dose by the phantom's radiosensitive organs. The TLD‐100 dosimeters presented the fattest energy‐dependent response compared to other TL pallet materials such as calcium sulfate doped with dysprosium (${\text{CaSO}}_{4}:\text{Dy}$), sintered alumina, TLD‐200, and TLD‐400. All other materials presented with a very high energy dependence of response for the attenuated beams, even for direct beams.^(17)^ The good results of TLD‐100 dosimeter in the energy‐dependent response tests is the main reason for its great popularity in dosimetry. In others circumstances, the TLD‐100 should be used and associated with a device that allows the effective energy of the beam to be estimated. The energy response is strongly dependent on the beam characteristics, such as total filtration, since there is significant variation of response for direct and attenuated beams in the same energy range. Therefore, the calibration curves should always be obtained at radiation qualities as close as possible in most aspects to those of clinical beams.^(17)^

The phantom was designed with a small fixed hole fitted for TLD placement precisely in each body organ. All 50 TLD chips were used in this study for measurement of the absorbed dose at the right and left thyroids (slab number 8), right and left breasts (slab number 15), esophagus (slab number 16), right and left lungs (slab number 16), liver (slab number 23), stomach (slab number 24), left kidney (slab number 25), right kidney (slab number 26), colon (slab number 28), gonad (slab number 34), urinary bladder (slab number 34), bone marrow (femoral head region) (slab number 34), and skin. For the skin dose measurements, the TLD chips were placed at the level of the seventh thoracic vertebrae (T7) (slab number 17) and iliac crest (slab number 28) for thoracic and abdomen–pelvic CT, respectively. Two TLD chips were used as a control to measure the background radiation. Each CT procedure (thoracic and abdomen–pelvic) was repeated three times with different TLDs, and the total TLD readings for each organ were averaged to calculate the dose for that organ. All TLD chips were kept for at least 24 hours before the readout process, using the Harshaw TLD Manual Reader Model 3500 (Thermo Electron Corporation, Solon, OH). This waiting time assured that any residual thermoluminescence from the short half‐life peaks would have no significant contribution on the signal.^18^, ^19^ The radiation dose may include exposure from the primary beam (the radiation beam targeted at the scanning range) and the secondary radiation beams (the scattered radiation dose produced from the primary beam), depending on the location of the organs.

Since the TLD reading was in nanocoulomb (nC) units, to convert the nC units into mGy, a calibration is needed. The TLD calibration was designed to create a graph pattern for radiation dose conversion from nC to mGy by exposing it to a known dose, which was measured with a digital radiation survey meter (model 660) having an ion‐chamber model 660‐3 beam measurement probe and a readout/logic unit, model 451P (Victoreen, Cleveland, OH).^(20)^ Sixteen TLD chips were exposed to a known radiation dose by a CT system with exposure parameters of 120 kVp and different mAs (10‐300 mAs) to approximate the radiation quality of a CT scanner.^(21)^ A correlation coefficient was then obtained based on the calibration value calculations.

#### B.2 Effective dose

The effective dose (ED) was used to provide a dose quantity that was related to the probability of health detriment due to stochastic effects resulting from exposure to low doses of ionizing radiation. It can be used to assess the risk of radiation‐induced cancers and serious hereditary effects to future generations, regardless of the procedure being performed, and is the most useful radiation dose indicator.^22^, ^23^ In fact, ED is derived from the weighted sum of doses to tissues that are known to be sensitive to radiation, and so can only be derived by calculation. The tissue weighting factors are derived from the extrapolation of epidemiological evidence. ED was intended for use in radiation protection, but it has been widely applied in the evaluation of doses for medical exposure involving only parts of the body.^(22)^

In this study, the ED was obtained by direct calculation of dose length product (DLP) and conversion coefficient (CC) based on the formula:

(1) ED=DLP×CC

in which the values of the conversion coefficient were used with reference to the International Commission on Radiological Protection (ICRP) publication 103.^(24)^ This conversion coefficient was derived from the body part anatomically specific to the region of the body being scanned in CT The conversion coefficient for thoracic and abdomen‐pelvic CT was $20.4\;\mu \text{Sv}\cdot {\text{mGy}}^{-1}\cdot {\text{cm}}^{-1}$ and $17.1\;\mu \text{Sv}\cdot {\text{mGy}}^{-1}\cdot {\text{cm}}^{-1}$ respectively.^(24)^ The DLP was available on the CT console.

#### B.3 Size‐specific dose estimate (SSDE)

The size‐specific dose estimate (SSDE) is defined as a patient dose estimate with corrections for the patient's size being taken into consideration before estimating the radiation dose from the volume CT dose index (${\text{CTDI}}_{\text{vol}}$) of radiation received by patients at each CT examination. The patient's size was measured using linear dimensions based on the CT images. Previously, the radiation dose estimated from ${\text{CTDI}}_{\text{vol}}$ was measured based on a 16 or 32 cm diameter polymethyl methacrylate (PMMA) cylindrical reference phantom, which is often called a ‘head’ or ‘body’ CTDI phantom, without taking into account appropriate patient size corrections. By using SSDE, the dose estimation is more accurate to some extent, and over‐ or underestimating radiation doses can be avoided, especially in pediatric CT scanning.^(25)^

Similar to the ED calculation, the SSDE was also obtained by direct calculation of the volume CT dose index (${\text{CTDI}}_{\text{vol}}$) with a conversion size factor ($f_{\text{size}}$) based on the formula:

(2) $$\text{SSDE}={\text{CTDI}}_{\text{vol}}\times f_{\text{size}}$$

in which the values of $f_{\text{size}}$ were used with reference to the American Association of Physicists in Medicine (AAPM) Report No. 204.^(25)^ Similar to DLP, the ${\text{CTDI}}_{\text{vol}}$ was recorded for each scan series, while the conversion factor was derived from the part anatomically specific to the region of the body being scanned with CT. Anteroposterior (AP) and lateral (LAT) dimensions at the seventh thoracic vertebrae level and iliac crest were measured from axial CT images using digital calipers on the scanner console. These measurements were summed to obtain the $f_{\text{size}}$, which was 1.45 for thoracic and 1.38 for abdomen‐pelvic, to represent patient size (AP+LAT). The $f_{\text{size}}$ values provided in the AAPM report were derived from the phantom's 32 cm diameter.

### C. Statistical analysis

The data were analyzed using Statistical Package for the Social Sciences (SPSS) V21.0 (SPSS version 21.0 for Windows). Continuous variables were expressed as median values±standard deviation. A p‐value of <0.05 was considered to indicate statistically significant differences. The Wilcoxon matched‐pairs signed‐rank test was used to analyze the multifactorial data, including ED, SSDE, and absorbed dose‐to‐radiosensitive organs for both the thoracic and abdomen–pelvic CT protocols with and without TCM. Further, Friedman's analysis of variance (ANOVA) was used in absorbed dose analysis to determine the differences in median absorbed dose between different radiosensitive organs from the two CT procedures for each protocol.

## III. RESULTS

The TLD calibration correlation coefficient was 0.1343 mGy/nC. For both the thoracic and abdomen–pelvic CT scans, the ED protocol differences with and without use of TCM were significant (p=0.003). The estimated ED was reduced significantly in the protocol with TCM, compared to that without TCM, for both the thoracic and abdomen–pelvic CT, with doses of 6.50±0.29mSv versus 20.07±0.24mSv and 6.01±0.20mSv versus 17.30±0.41mSv, respectively (Table 2).

The SSDE also showed a significant difference between protocols with and without TCM for both the thoracic and abdomen–pelvic CT scans (p=0.001). Similar to the findings with ED, the SSDE was also reduced significantly in the protocol with TCM, compared to that without TCM, for both the thoracic and abdomen–pelvic CT, with doses of 10.18±0.48mGy versus 31.56±0.43mGy and 11.96±0.27mGy versus 33.23±0.05mGy, respectively (Table 2).

**Figure 2:**
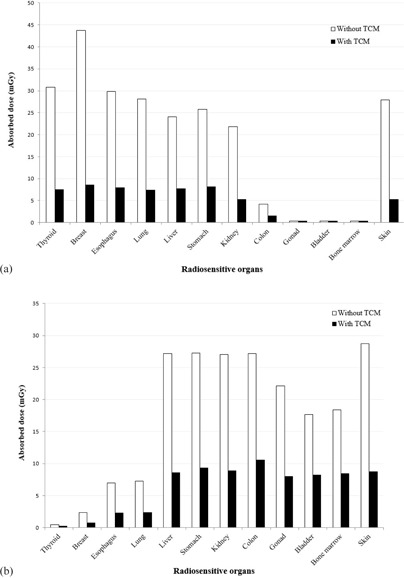
Absorbed dose of radiosensitive organs: (a) from the thoracic CT examination. The graph shows that the breast received the highest dose in both the protocols with and without TCM compared to that received by other radiosensitive organs during the thoracic CT procedure. In fact, the breast absorbed approximately four times more when no TCM protocol was used. Absorbed dose of radiosensitive organs: (b) from the abdomen‐pelvic CT examination. The graph shows that without TCM, the skin absorbed the most radiation dose during the abdomen–pelvic CT procedure. However, with TCM, the highest absorbed dose was reported at the colon. In this CT examination, the thyroid absorbed the lowest radiation dose in both protocols with and without TCM.

The radiation dose for each organ was measured to compare the CT scanning protocols with and without TCM. The dose was quantified as an entire dose for bilateral organs (the thyroid, breasts, lungs, and kidneys). The absorbed dose measured at radiosensitive organs in thoracic and abdomen–pelvic CT protocols did not differ significantly for the protocols with TCM and without TCM. The breast received the highest absorbed dose in the thoracic CT both with and without TCM, with dose values of 8.58±0.12mGy and 51.52±14.72mGy, respectively (Fig. 2(a)). On the other hand, the absorbed dose was the lowest for the bone marrow, which received doses of 0.29±0.04mGy and 0.36±0.02mGy in the protocols with and without TCM, respectively.

In the abdomen–pelvic CT examination, the highest absorbed dose was received by the skin at 9.30±1.28mGy and 29.99±2.23mGy for the protocols with and without TCM, respectively (Fig. 2(b)). The thyroid received the lowest absorbed radiation dose during abdomen–pelvic CT, which was reported at 0.23±0.01mGy and 0.46±0.05mGy for the protocols with and without TCM, respectively.

**Table 2:** Mean radiation doses from thoracic and abdomen–pelvic CT

	*Thoracic CT*		*Abdomen–Pelvic CT*	
*Radiation Doses*	*With TCM*	*Without TCM*	*With TCM*	*Without TCM*
ED (mSv)	6.50±0.29	20.07±0.24	6.01±0.20	17.30±0.41
SSDE (mGy)	10.18±0.48	31.56±0.43	11.96±0.27	33.23±0.05

ED=effective dose,SSDE=size‐specific dose estimate,TCM=tube current modulation.

## IV. DISCUSSION

Three important findings flow from this study. First, with use of TCM in thoracic and abdomen–pelvic CT scans, the radiation doses in terms of ED and SSDE can be reduced significantly by up to 68%. Second, the highest and lowest absorbed doses were received by the breast and bone marrow, respectively, in the thoracic CT, and by the skin and thyroid, respectively, in the abdomen–pelvic CT. Third, the absorbed dose measured at the radiosensitive organs in both the thoracic and abdomen–pelvic CT were reduced with significant differences between protocols with and without use of TCM.

The ED and SSDE results were significantly higher in protocols without TCM for both CT examinations (thoracic and abdomen–pelvic) because of the tube current remaining constant for all scan lengths. By using TCM, 68% and 63% of ED reduction can be achieved in thoracic and abdomen–pelvic CT, respectively. This result was supported by a previous study in which it was shown that TCM can reduce ED by 11%.^(26)^ Similarly, dose reduction in SSDE can be achieved when using TCM by 64%–68% for both thoracic and abdomen–pelvic CT.^(27)^

For both the TCM protocol and the protocol without TCM, the thoracic CT was estimated to receive a higher ED compared to that received from the abdomen–pelvic CT. The results were inconsistent due to the material used in RANDO phantom which was designed for megavoltage application suitable for radiotherapy, but not for diagnostic energy range purposes.

However, the scan length of the thoracic CT is shorter than that of abdomen–pelvic CT; the lengths were 389 mm and 410 mm, respectively. Hence, the higher dose from the thoracic CT may be due to slice width in the thoracic CT, which is narrower than that in the abdomen–pelvic CT; the widths were 5 mm and 10 mm, respectively. When the slice thickness is reduced, the size (volume) of the individual tissue volume element (called voxels) is reduced, thus resulting in a decrease of absorbing or capturing radiation or in the number of X‐ray photons.

In contrast, SSDE yielded different results, with the abdomen–pelvic CT receiving a higher SSDE than the thoracic CT. The thoracic region has a smaller body thickness compared to the abdomen–pelvic region and, therefore, should require a much lower exposure factor setting compared to that in the abdomen–pelvic CT. The evidence for this was reflected in the measurement of the phantom's AP+LAT during the conversion factor of the SSDE, in which the abdomen–pelvic region has a larger size compared to that in the thoracic region. As the SSDE greatly depends on the patient's body size, any increase of size or diameter of the scanning region will accordingly contribute a significant change in the SSDE.

Although the ED calculation depends on the ICRP and ICRU weighting factors for the internal organs, ED is another way to estimate the radiation dose received by patients during CT examinations, since the absorbed dose has to be estimated based on ${\text{CTDI}}_{\text{vol}}$ values. The exact figure for the absorbed radiation dose received by patients remains debatable. Measuring absorbed doses using TLD devices is regarded as the gold standard in CT dose quantification. However, this technique is not clinically practical due to limitations of the TLD chips placed inside the patient's body and, in addition, the technique is time‐consuming. However, several studies have reported that the patient dose can be quantified by using SSDE, which is a great step forward in monitoring and controlling the CT imaging radiation dose.^(28)^ SSDE is defined as patient dose estimation in mGy based on the ${\text{CTDI}}_{\text{vol}}$ value, with a correction factor for the patient's size taken into consideration. The patient's linear dimensions are obtained by measuring the AP+LAT diameter on real patients or patient images.^(25)^ It was first introduced by developing conversion factors that take into account patient size, and hence is especially important for pediatric CT and scanning small body sizes.^(25)^ It helps to ensure that patients with a smaller diameter (pediatric) do not receive a high dose during CT examinations. Besides, by using SSDE, practitioners are able to estimate the patient dose associated with a specific size by using the effective diameter of the patient's body, which corresponds to a circle having an area equal to the patient's cross section in CT images.^(25)^ Furthermore, the SSDE measurement can be used for a range of patient sizes (for large and small patients) for estimation of doses from CT examinations.^29^, ^30^, ^31^

In each CT examination, the absorbed doses of the organs were found to differ significantly. This is due to location of the radiosensitive organ — whether it is located at the area of primary radiation or scattered radiation. For example, in a thoracic CT scan, the absorbed doses of the breast as compared to those of the gonad, bladder, and bone marrow differ significantly. This is because the gonad, bladder, and bone marrow are located within the scattered radiation area as opposed to the breast, which is within the primary radiation area. In thoracic CT, the phantom was positioned at the center of the X‐ray tube so that the radiosensitive organs lie in the thoracic region, such as the breast, with the most centered organs receiving the maximum dose from the primary radiation beam. However, the thyroid and gonad only received scattered radiation since they were anatomically situated adjacent to the thoracic region. This was verified by the TLD measurement in our study. These findings support those of previous studies in which the dose received by particular organs was highly dependent on their anatomical location and distance from the primary radiation area. Specifically, the organs situated within the scattered radiation area were likely to absorb secondary radiation, hence receiving a much lower dose.^20^, ^32^, ^33^

There are some limitations to this study. First, the study used an adult phantom to simulate the radiation dose that would be received by a standardized patient, and no contrast medium was introduced, so the image quality could not be assessed. Thus, the radiation dose measurements were carried out without image quality evaluation. Therefore, further studies are necessary to verify the accuracy of the results with both qualitative and quantitative assessments of radiation dose and image quality. In addition, only one scanner from Siemens was used for this study; hence, only one tube current modulation system was evaluated. Since the main limitation of automatic TCM is a lack of uniformity between techniques developed by different vendors, studies using TCM systems from different vendors are, therefore, recommended to further evaluate significant dose reduction with this technique.

## V. CONCLUSIONS

The TCM technique can significantly reduce the radiation dose received by patients during thoracic and abdomen–pelvic CT in terms of effective dose and size‐specific dose estimates. Results for the absorbed dose of the individual organs reveal significant difference between CT with TCM and without TCM, so that the TCM technique should be used to help reduce the absorbed dose received by the radiosensitive organs. Thus, this technique is highly recommended to further reduce the radiation dose that patients receive.

## ACKNOWLEDGMENTS

We would like to thank Research University Grant (GUP‐2014‐053), Universiti Kebangsaan Malaysia, for financial support of the project. The author wish to thanks Mr. Fakhrur Razi Kufian, Mr. Mohd Nizar Mohd Mokhtar, Ms. Norjana A Rahman, and Ms. Hafizah A. Rashid from Diagnostic Imaging & Radiotherapy Program, PPSDKG UKM and Mr. Mohd Norman Mohd Nordin from CT unit, UKM Medical Centre, for their assistance in data collection.

## Supporting information

Supplementary Material

Supplementary Material
