# Supplementary figures and images for: Radiation dose reduction in thoracic and abdomen–pelvic CT using tube current modulation: a phantom study
Source: J Appl Clin Med Phys. 2014 Jan 8;16(1):319–28. doi: 10.1120/jacmp.v16i1.5135 (PMC5689995; doi:10.1120/jacmp.v16i1.5135)

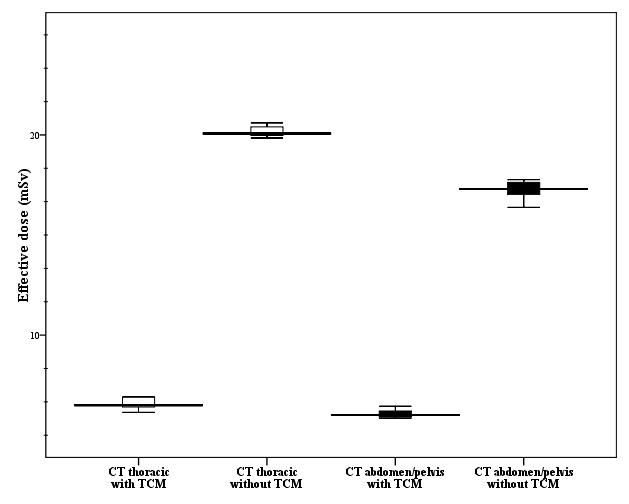

Supplement: Supplementary file 1 — Supplementary Material [file ACM2-16-319-s001.jpg]

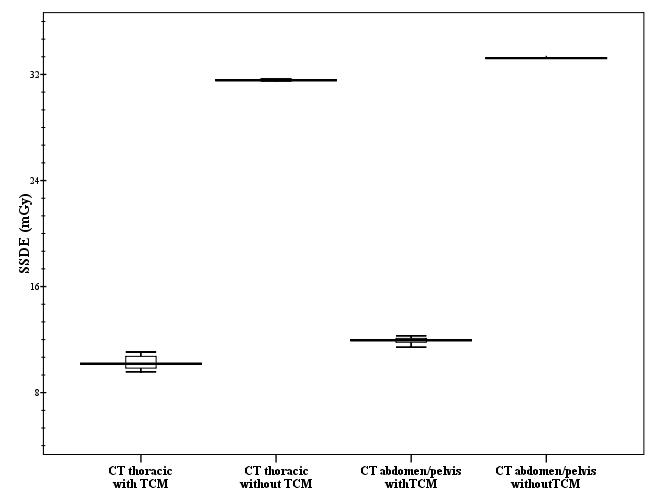

Supplement: Supplementary file 2 — Supplementary Material [file ACM2-16-319-s002.jpg]
